# Glial cell interactions and glaucoma

**DOI:** 10.1097/ICU.0000000000000125

**Published:** 2015-02-05

**Authors:** Rachel S. Chong, Keith R. Martin

**Affiliations:** a John van Geest Centre for Brain Repair, University of Cambridge; b Wellcome Trust-Medical Research Council Cambridge Stem Cell Institute; c Cambridge NIHR Biomedical Research Centre; d Eye Department, Addenbrooke's Hospital, Cambridge, UK

**Keywords:** glaucoma, glia, monocyte, neuroprotection

## Abstract

**Purpose of review:**

The present review describes new advances in our understanding of the role of glial cells in the pathogenesis of glaucoma. It is becoming clear that retinal glia should not be studied in isolation in glaucoma because glia have dynamic and diverse interactions with a range of different cell types that could influence the disease process.

**Recent findings:**

Microglial activity is modulated by signals from retinal ganglion cells and macroglia that influence RGC survival in various models of injury. New studies suggest that circulating monocytic populations may play a role in mediating the immune response to glaucoma. Astrocytes have been found to develop discrete localized processes that interact with a specific subset of retinal ganglion cells, possibly responding to the expression of phagocytic signals by stressed retinal ganglion cells.

**Summary:**

Retinal glia constitute a highly versatile population that interacts with various cells to maintain homeostasis and limit disease. Defining the mechanisms that underlie glial communication could enable the development of more selective therapeutic targets, with great potential clinical applications.

## INTRODUCTION

Glaucoma is an optic neuropathy that predominantly affects retinal ganglion cells (RGCs), although evidence is accumulating that glial cells in the retina also play a major role in the pathogenesis of this important cause of irreversible blindness. Retinal glia consist of several populations of multifunctional, highly adaptable cells that mediate normal retinal function through close relations with a variety of different cell types. The importance of these interactions and new insight into the possible ways in which they influence our understanding of glaucoma will be discussed in the present review.  

**Box 1:**
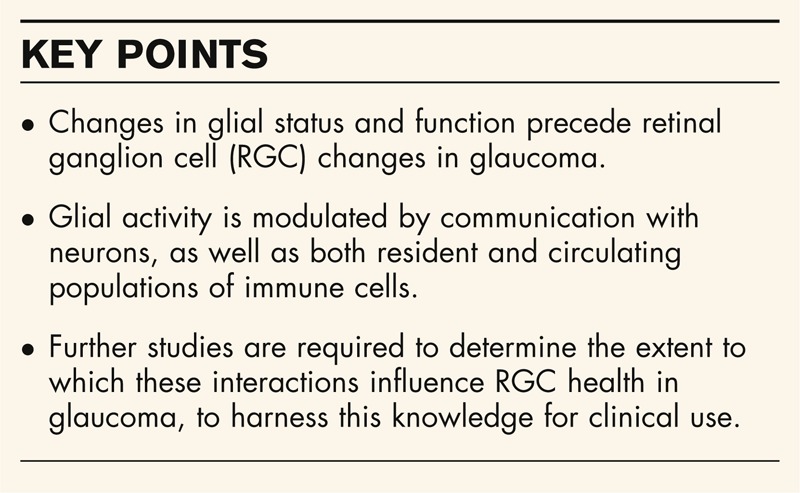
no caption available

## MICROGLIA AND MONOCYTE-DERIVED MACROPHAGES IN THE RETINA

Microglia are resident immune cells normally found in the central nervous system (CNS) and retina that play an essential role in the innate immune response. Microglia were first described by del Rio-Hortega in 1932 as the nonneuronal, nonastrocytic ‘third element of the CNS’ and show considerable homology with markers for monocytes and monocyte-derived macrophages including F4/80, CD11b and Iba-1 [1,2]. Together with astrocytes and Müller glia, they help to maintain homeostasis in the retina through regulating ion exchange, glucose and neurotransmitter transport. Microglia also have an important function as highly specialized ‘policing cells’ that continuously survey the microenvironment and quickly respond to neuronal injury by phagocytosing potentially harmful neuronal debris to limit damage, secreting local inflammatory mediators and communicating with other potential immune effector cells [3]. The transformation of surveying microglia into alerted or reactive states, in response to a perceived threat, is triggered by signals from other components of the immune system, as well as the expression of neuronal ligands such as fractalkine (CX3CL1) or CD200 [4^▪^].

Microglia are thought to arise from yolk sac macrophages that enter the primitive brain during early fetal development, although it is now believed that circulating monocytes may also subsequently invade the CNS and differentiate into microglia in the early postnatal stages [5^▪▪^]. In adults, there appears to be a clear distinction between bone marrow-derived cells and the resident microglial population under normal conditions in which the blood–brain barrier has not been breached [6]. Under certain inflammatory conditions, however, including neurodegenerative diseases with an underlying inflammatory disorder such as multiple sclerosis or Alzheimer's disease, bone marrow-derived circulating progenitor cells may supplement resident microglial populations [7,8].

Our perspective on the importance of microglia in glaucoma is gradually becoming clearer. Studies using the DBA/2J mouse model of glaucoma, which are an inbred strain of mice that spontaneously develop progressive glaucoma, suggest that changes to the location and activation status of microglia occur at the early stages of the disease, preceding detectable RGC disease [9]. Other studies in which translimbal trabecular laser photocoagulation was used to induce intraocular pressure (IOP) elevation in rats have also shown that elevated IOP increases both microglial activity and density in the retina, optic nerve and tract before any sign of RGC axonal loss [10]. Microglial in the laminar zone between the optic nerve head (ONH) and myelinated optic nerve show the earliest changes in DBA/2J mice, corresponding to the location where axonal damage is first thought to occur [11].

It is possible that an increase in microglial proliferation and activation could have a detrimental effect on RGCs via secretion of proinflammatory cytokines such as interleukin-6, tumour necrosis factor-alpha (TNF-α) and reactive oxygen species [3]. Consistent with this theory, inactivation of microglia using minocycline seems to have a neuroprotective effect on RGCs in DBA/2J mice [12]. Deficiencies in the chemokine fractalkine, which is produced by neurones and moderates microglial activity under normal conditions, have also been found to be associated with increased RGC loss in an experimental mouse model of glaucoma [13^▪▪^]. The present study provides the first evidence of RGC-mediated dampening of microglial reactivity under normal circumstances, and that disruption of this signalling pathway exacerbates RGC death because of glaucoma.

At present, the role of infiltrating monocyte-derived macrophages in glaucoma is less certain. Circulating monocytes were seen to invade the retina and optic nerve in aged glaucomatous DBA/2J mice, whereas ocular irradiation that reduced the presence of these cells resulted in an enhanced RGC survival [14]. Increasing the number of monocytes in the peripheral vasculature, however, was also reported to have a protective effect in mice that experienced high IOP secondary to anterior chamber cannulation [15]. These differences in the RGC response to monocyte-derived macrophages in the glaucomatous eye are still not well understood, but may well relate to specific aspects of the individual disease models that have yet to be defined.

Retinal glia play an important part in maintaining synaptic activity in the retina by transporting neurotransmitters and preserving biochemical homeostasis through modulation of ion channel distribution. Microglia in particular seem to have a unique involvement with retinal synapses, as demonstrated by studies that show recurring intimate contact between their filopodia and dendritic spines [16]. Complement factor C1q is also profoundly upregulated on RGC synapses with increasing severity of glaucoma in DBA/2J mice. This is thought to present a target for subsequent elimination by microglial engulfment [17]. Whether this process of synaptic stripping serves the purpose of limiting inflammatory debris, or, conversely, represents a dysfunctional state that contributes to RGC death, remains to be seen.

## MÜLLER CELLS

Other important glial cell populations in the eye include astrocytes and Müller cells. Müller cells are the principal macroglial cells found in the retina and are radially oriented cells spanning the entire thickness of the retina. The Müller cell body lies in the inner nuclear layer and elongates into two main trunks that pass towards the inner limiting membrane overlying the RGC/retinal nerve fibre layer as well as to the outer limiting membrane directly adjacent to photoreceptors. Müller cells are characterized by a broad plate known as the Müller endfoot adjacent to the inner limiting membrane and by apical microvilli close to the photoreceptor layer. Numerous processes protrude from the main trunks of Müller cells, which interact with various neuronal cell types in the different layers of the retina.

Müller cells are thought to play an essential role in maintaining the structural integrity of the retina and are also important regulators of retinal cell metabolism. They act as an anatomical conduit between retinal neurones and the cellular environment, maintaining retinal homeostasis by participating in essential processes such as glucose metabolism, antioxidant production, ion/substrate exchange and vascular regulation. A newer idea about the unique properties of Müller cells is that they may also act as light-guiding fibres because of their structure and specific refractive index, relative to the inner and outer retinal surfaces that they bridge [18].

Müller cells moderate neurotransmitter levels by providing precursor substrates to neurones and preventing toxic accumulation of glycine, gamma-aminobutyric acid and glutamate in the retina [19] that could otherwise lead to the activation of proapoptotic signalling in neurones [20]. Müller cells also promote RGC survival through the production of neurotrophic factors such as ciliary neurotrophic factor, which has been shown to have potent neuroprotective effects in glaucoma [21].

In glaucoma, the activation of Müller cells also plays a crucial role in driving microglial migration and the recruitment of other immune cells within the retina [22]. The combined actions of astrocytes and Müller cells provide a supportive environment for the modulation of synaptic activity through the activation of microglia and the maintenance of ionic and neurotransmitter levels [23]. New evidence suggests that upregulation of microglial–macroglial cross-talk occurs in response to optic nerve axotomy or excitotoxic injury to the retina, which could serve to modulate microglial activity to promote a return to baseline quiescence [24^▪▪^]. Müller cells may also form an adhesive cellular scaffold that guides the movement of microglia across various retinal layers to mount an effective response to injury [25].

Recent studies of the biomechanical properties of Müller cells have shown that, perhaps surprisingly, they are softer than neurones, challenging the concept of a ‘hard’ scaffold for retinal cells [26]. This interesting feature of Müller cells suggests that they may provide cushioning support that helps to resist physical forces acting on the retina, such as traumatic injury or raised IOP. It is also possible that a soft, pliant environment provides a more conducive environment for neurite outgrowth. In glaucoma, Müller cells undergo similar reactive changes that mirror the response described in astrocytes at the ONH with hypertrophy and formation of a glial scar in rodent models of the disease [27]. Furthermore, the upregulation of intermediate filaments such as nestin, vimentin and glial fibrillary acidic protein (GFAP) may increase the stiffness of these cells, reversing the benefits of the ‘soft’ perineuronal environment described above [28,29].

## ASTROCYTES

Astrocytes are the major glial cell type found at the ONH. These cells line the lamina cribrosa pores and blood vessels to form a dense network that resembles a honeycomb spanning the ONH. RGC axons passing through the lamina cribrosa are organized into bundles interspersed between astrocytic processes, which provide support by secreting extracellular matrix molecules and also form part of the blood–retinal barrier.

In several models of glaucoma, astrocytes appear to demonstrate increased GFAP immunoreactivity as well as extracellular matrix remodelling at the ONH. GFAP is an intermediate filament of the glial cell cytoskeleton, and becomes increasingly expressed during the transformation of mature, quiescent astrocytes into their ‘reactive’ state. It has been known for some time that astrocytes in the glaucomatous lamina cribrosa undergo morphological changes that mimic the CNS injury response, characterized by hypertrophy of the cell body, and a generalized loss of thick processes [30]. More recent studies of cross-bred mouse strains that develop spontaneous glaucoma in addition to having GFP-expressing astrocytes have also demonstrated the presence of long, thin, non-GFAP-expressing processes in astrocytes responding to high IOP. These fine processes that project into the axon bundle appear to be confined to specific regions of each astrocyte [31^▪^] and may only be in contact with several RGC axons at each point, instead of spanning across the diameter of the optic nerve.

The precise function of these fine processes is still unknown, but it is possible that they have a limited structural role because they do not make up part of the glial tubes that surround RGC axon bundles. Instead, they may mediate local inflammatory responses through TNF-α/TNF-receptor signalling and nuclear factor-κB activation, and regulate autophagy via the serine/threonine protein kinase mammalian target of rapamycin pathway [32]. This segregated pattern of astrocyte–RGC interaction could explain the sectorial degeneration of RGC axons, which is typically seen in glaucoma.

Another interesting possibility is that these processes may have phagocytic properties that act on damaged RGCs. Astrocytes within the postlaminar ONH myelination transition zone of healthy eyes have recently been found to express the phagocytosis-related gene *Mac-2*[33]. Expression of this gene becomes upregulated in glaucoma in astrocytes of the laminar and orbital regions of the optic nerve, suggesting that certain astrocytes could have intrinsic phagocytic abilities that aid in the clearance of harmful RGC axonal debris. Neurones are known to constitutively express ‘eat-me’ or ‘do-not-eat-me’ signals in the CNS [34^▪▪^], which may be mirrored in RGCs to regulate this phagocytic response.

In addition to their location at the ONH, astrocytes are also found spread out across the retinal nerve fibre layer (RNFL) where they form a network of spindle-shaped cells with close associations with retinal vasculature. A rodent experimental model of glaucoma has suggested a generalized loss of astrocyte coverage over the RNFL, and subtle morphological changes were seen in both the injured and the uninjured eyes of the same animal, although the reason for this ‘sympathetic’ response seen in the fellow retina is still unknown [35].

Astrocytic dysregulation of vascular permeability and endothelial cell activation is also increasingly thought to be a prominent feature of glaucoma. In addition to the reports of enhanced transendothelial monocyte migration seen in experimental models of glaucoma described earlier, patients with primary open-angle glaucoma have been observed to have increased vascular permeability at the optic disc using fluorescein angiography [36]. A postmortem veterinary study of dogs who experienced goniodysgenesis and primary glaucoma showed evidence that suggests a compromised blood–retinal barrier in diseased eyes as well [37].

Paradoxically, or perhaps in response to the breakdown of the blood–retinal barrier, reactive ONH astrocytes have also been found to express increased levels of endothelin-1 (ET-1) receptors in experimental glaucoma [38]. ETs belong to a family of potent vasoconstrictive molecules that are produced by astrocytes in response to stretch [39] and act in a paracrine loop on ET receptors to cause proliferation and activation of astrocytes, in addition to impeding ocular blood circulation [40]. Experimental glaucoma models of both rat and DBA/2J mice have been demonstrated to have increased expression of ET-1 correlating with neuronal loss [41,42]. Elevated levels of ET-1 have also been found in the plasma and aqueous humour of glaucoma patients, suggesting that these molecules may indeed have a potential involvement in the human disease [43].

## CONCLUSION

Recent studies have provided increasing evidence that retinal glia do not perform isolated functions but are engaged in various important relations with other cells in the retina relevant to optic nerve diseases such as glaucoma. These interactions are not limited to neuron–glia communication, but also extend to endothelial cells that line blood vessels, circulating cells from the peripheral blood supply and possibly even the contralateral eye.

Certainly, modulation of homeostatic processes that favour neuroprotection over damaging chronic inflammation appears to be a key factor in mediating the consequences of glial activity. Cross-talk between microglia and RGCs as well as other types of retinal glia may be pivotal in managing the delicate balance of ‘friend or foe’ cues that determine glial behaviour in the presence of disease. Therapeutic strategies that selectively target certain aspects of glial signalling in the retina could have a great potential for future clinical applications.

In conclusion, a deeper understanding of the complex network of glial interactions in the retina would undoubtedly produce much insight into the pathogenesis of glaucoma.

## Acknowledgements

None.

### Financial support and sponsorship

The authors were supported by the Agency for Science, Technology and Research (Singapore), Fight for Sight (UK), the Cambridge Eye Trust (UK) and the Jukes Glaucoma Research Fund (UK).

### Conflicts of interest

There are no conflicts of interest.

## REFERENCES AND RECOMMENDED READING

Papers of particular interest, published within the annual period of review, have been highlighted as:

▪ of special interest
▪▪ of outstanding interest
